# Safety, pharmacokinetics and efficacy of SCT200, an anti-EGFR monoclonal antibody in patients with wild-type *KRAS/NRAS/BRAF* metastatic colorectal cancer: a phase I dose-escalation and dose-expansion study

**DOI:** 10.1186/s12885-022-10147-9

**Published:** 2022-10-28

**Authors:** Wen Zhang, Xiaohong Han, Lin Yang, Yuanyuan Song, Liangzhi Xie, Wenlin Gai, Yan Wang, Yuankai Shi

**Affiliations:** 1 Department of Medical Oncology, National Cancer Center/National Clinical Research Center for Cancer/Cancer Hospital, Chinese Academy of Medical Sciences & Peking Union Medical College, Beijing Key Laboratory of Clinical Study on Anticancer Molecular Targeted Drugs, Beijing, China; 2 Clinical Pharmacology Research Center, Peking Union Medical College Hospital, State Key Laboratory of Complex Severe and Rare Diseases, NMPA Key Laboratory for Clinical Research and Evaluation of Drug, Beijing Key Laboratory of Clinical PK & PD Investigation for Innovative Drugs, Chinese Academy of Medical Sciences & Peking Union Medical College, Beijing, China; 3 Beijing Engineering Research Center of Protein and Antibody, Sinocelltech Ltd, Beijing, China

**Keywords:** Monoclonal antibodies, Colorectal cancer, Epidermal Growth Factor Receptor, SCT200

## Abstract

**Background:**

An over-expression of the epidermal growth factor receptor (EGFR) has been observed in colorectal cancer and is associated with aggressive disease and poor prognosis. SCT200 is a newly developed recombinant, fully humanized, anti-EGFR monoclonal antibody. This study aimed to evaluate its safety, tolerability, pharmacokinetics (PK), and efficacy in patients with wild-type *KRAS/NRAS/BRAF* metastatic colorectal cancer (mCRC).

**Methods:**

This phase I study comprising dose-escalation phase and dose-expansion phase. SCT200 was administrated intravenously to groups of three to six patients. An every 3-week dosing cycle (0.5–15.0 mg/kg) and multiple dosing schedule were evaluated. Blood samples were collected at preset intervals for PK assessment, radiological imaging was used for efficacy assessment, and continuous safety monitoring was performed in each group during the study.

**Results:**

From December 16, 2014 to December 31, 2018, fifty-six patients with wild-type *KRAS/NRAS/BRAF* mCRC receiving ≥ 1 dose of SCT200 were evaluated. Among them, 44.6% (25/56) of the patients failed at least two prior lines of chemotherapy. No dose-limiting toxicities occurred in any group. All of the patients experienced treatment-emergent adverse events (TEAEs). 96.4% (54/56) of patients experienced treatment-related adverse events (TRAEs), and 26.8% (15/56) of patients with Grade ≥ 3 TRAEs. No serious TRAEs were observed. The most common TRAEs were dermotoxicity and hypomagnesemia. PK analysis showed non-linear PK in the range of 0.5 - 8.0 mg/kg of single dose SCT200, the clearance decreased, and the elimination half-life (T_1/2_) prolonged following dose increase. In the multiple-dose period, the clearance decreased, peak concentration increased, and T_1/2_ prolonged during prolonged drug administration, and a steady state was reached after five consecutive dose of 6.0 mg/kg quaque week (QW). The objective response rate (ORR) was 30.4% (17/56, 95% confidence interval [CI], 18.8%–44.1%). The ORR in the dose-expansion group (6.0 mg/kg QW) was 48.0% (12/25, 95% CI, 27.8%–68.7%), the median progression-free survival was 5.2 months (95%CI, 3.6–5.5), and the median overall survival was 20.2 months (95%CI, 12.1-not reached).

**Conclusions:**

SCT200 showed favorable safety, PK profile, and preliminary efficacy for patients with wild-type *KRAS/NRAS/BRAF* mCRC.

**Trial registration:**

This study was registered with ClinicalTrials.gov (NCT02211443).

**Supplementary Information:**

The online version contains supplementary material available at 10.1186/s12885-022-10147-9.

## Background

The epidermal growth factor receptor (EGFR) pathway plays a key role in tumorigenesis, cancer cell survival, migration, angiogenesis and apoptosis. EGFR overexpression has been found in 50–80% patients of colorectal cancer (CRC) and its increased levels are associated with aggressive disease and poor prognosis [1, 2]. Among the anti-EGFR monoclonal antibodies (cetuximab, panitumumab, nimotuzumab, and necitumumab) used in cancer treatment, cetuximab and panitumumab have been approved for the treatment of metastatic CRC (mCRC). Cetuximab or panitumumab in combination with chemotherapy have been established as standard first-line regimens for the treatment of wild-type *KRAS/NRAS/BRAF* mCRC, which extended the overall survival (OS) by 6 to 8 months over chemotherapy alone [1–7].

SCT200 is a fully humanized anti-EGFR monoclonal antibody which was developed by Sinocelltech Ltd., Beijing, China, with an antigen-binding epitope, physicochemical properties, and biological activity that are different from those of currently marketed anti-EGFR monoclonal antibodies. The binding affinity to EGFR (Kd = 0.08 nM) of SCT200 is comparable to that of panitumumab (Kd = 0.05 nM), and higher than that of cetuximab (Kd = 0.147 nM) and nimotuzumab (Kd = 1 nM). The tumor-targeted monoclonal antibodies may increase the anticancer effects through antibody-dependent cellular cytotoxicity (ADCC). For this purpose, SCT200 is specifically designed to enhance ADCC and complement-dependent cytotoxicity (CDC) through the Fc domain, which exhibits ADCC mediated anticancer activity at low concentration. Preclinical studies showed that SCT200 alone significantly inhibited the growth of vulvar squamous cell carcinoma and colon cancer cells in vivo, and the anticancer activity was enhanced when combining with chemotherapeutic agents (data unpublished). Comparing with cetuximab, SCT200 demonstrated superior inhibition of tumor cell growth in vitro and in vivo. The target organs of toxicity of SCT200 were mainly the skin and gastrointestinal system, and there were no non-target-related toxic effects observed in the preclinical study (data unpublished).

This is the phase I, dose-escalation and dose-expansion study to investigate the safety, tolerability, pharmacokinetics (PK), and efficacy of SCT200 in patients with wild-type *KRAS/NRAS/BRAF* mCRC who had failed prior chemotherapies (NCT02211443).

## Patients and methods

### Patient eligibility

Patients aged 18–70 years with pathologically confirmed CRC who had prior treatment failure with fluorouracil/oxaliplatin/irinotecan, wild-type *KRAS/NRAS/BRAF*, measurable/nonmeasurable tumor lesions, an Eastern Cooperative Oncology Group (ECOG) performance status (PS) of 0–1 and life expectancy over 3 months, with adequate organ functions and no history of other malignancies were enrolled. The main exclusion criteria were: prior treatment with EGFR inhibitors, anticancer therapy or surgery within 4 weeks, Grade ≥ 2 toxicity due to prior anticancer therapy, and any other condition deemed inappropriate by the investigator. The mutation status (exons 2, 3 and 4 for both *KRAS* and *NRAS*, and *BRAF V600E*) of all patients were examined in the screening period, and patients with *RAS* (*KRAS/NRAS* 2, 3 and 4 exons) and *BRAF V600E *mutation were excluded from the study. The detailed study protocol Synopsis was presented in Supplementary file 1.

This study was approved by the Ethics Committee of the National Cancer Center/Cancer Hospital, Chinese Academy of Medical Sciences & Peking Union Medical College (approval number: 14–057/ 847), and was conducted in accordance with the Declaration of Helsinki and Good Clinical Practice guidelines. All patients provided written informed consent.

### Study design

**Fig. 1:**
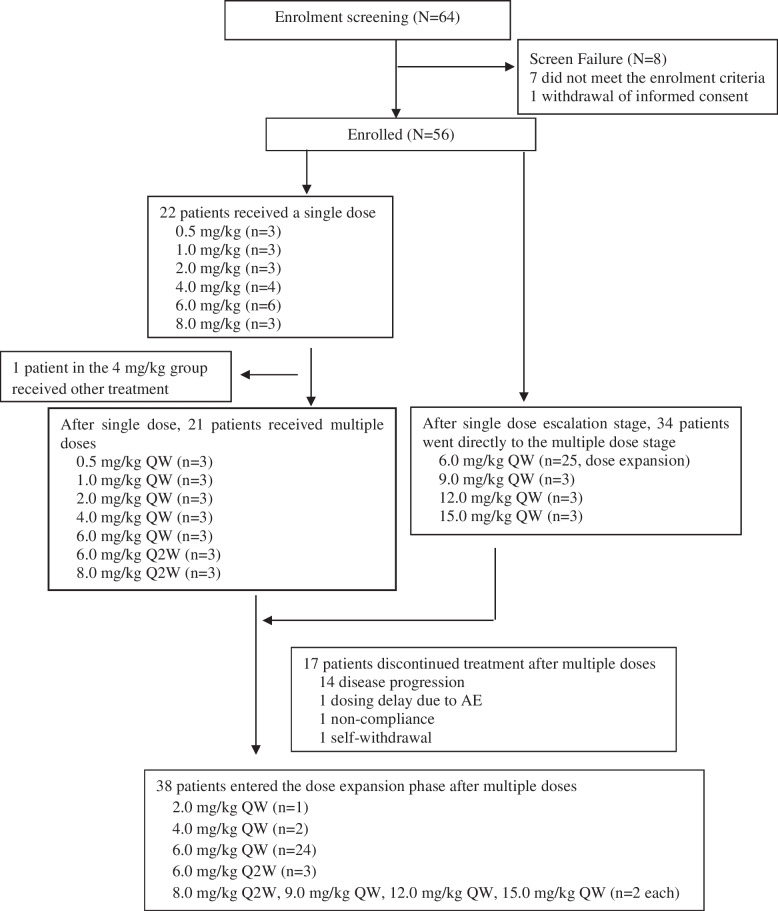
Phase I trial flowchart. AE adverse event, QW Quaque week, Q2W Quaque 2 week

The study was composed of the dose-escalation phase and dose-expansion phase. SCT200 was administrated intravenously to groups of three to six patients. The starting dose and the maximum dose of SCT200 was set as 0.5 mg/kg and 15.0 mg/kg, respectively based on the results of preclinical studies. Patients in the six low-dose escalation groups (0.5 mg/kg, 1.0 mg/kg, 2.0 mg/kg, 4.0 mg/kg, 6.0 mg/kg, and 8.0 mg/kg) first received a single ascending dose (SAD), were monitored for 3 weeks, and then entered the multiple ascending dose (MAD) stage. In the MAD stage, patients in 0.5 mg/kg, 1.0 mg/kg, 2.0 mg/kg, and 4.0 mg/kg groups were administered SCT200 once per week for 4 weeks. In the 6.0 mg/kg group, three patients were administered one dose of SCT200 per week for 6 weeks and another three patients received one dose of SCT200 in 2 week intervals for 6 weeks. The 8.0 mg/kg group was administered with SCT200 once in 2 weeks for 6 weeks. Patients in three high-dose MAD groups (9.0 mg/kg, 12.0 mg/kg, and 15.0 mg/kg) were administered one dose of SCT200 per week for 6 weeks. SAD/MAD of the next dose group were started on the premise that the dose level of SCT200 on the previous dose group was safely tolerated in a 3-week observation period. In addition, a dose-expansion phase for evaluating a target dose of SCT200 was initiated when the dose escalation phase was completed. The target dose was determined based on the PK analysis of the dose escalation phase, and a total of 25 patients were administered one dose of SCT200 at the target level per week for 6 weeks. After the completion of the pre-specified multiple dosing cycles, if the tumor was in remission or stable, patients were allowed to continue the treatment of SCT200 at the respective dose levels until disease progression or intolerable toxicity occurred (Fig. 1). The dose-limiting toxicity (DLT) was defined as (1) Grade 3/4 adverse events (AEs) (other than Grade 3 rash) that were related to SCT200, based on the judgment of the investigator; (2) any serious/life-threatening AEs that were not listed in the National Cancer Institute Common Terminology Criteria for Adverse Events (CTCAE) version 4.0, which was deemed by the investigator to be related to SCT200; (3) Grade 3 rash, if the following serious dermotoxicities were present: desquamation, extensive urticaria, symptomatic dermotoxicity requiring pharmacologic intervention (analgesics/corticosteroids), and dermotoxicity that was intolerable to the patient; and (4) any interruption of two scheduled infusions owing to Grade 3 dermotoxicity.

### Assessments

Safety and efficacy evaluations were performed on all patients who received ≥ one dose of SCT200 treatment. Safety assessments were performed at baseline, during the 21-day single-dose period, before and once a month after entering the multiple-dose period. CTCAE version 4.0 was used to determine the severity of AEs. Tumor response was assessed according to the Response Evaluation Criteria in Solid Tumors (RECIST) version 1.1 at baseline, the end of the multiple-dose treatment cycle, and every 8 weeks after entering the multiple-dose expansion period until disease progression (PD) or intolerable toxicity occurred. Objective response rate (ORR) was defined as the percentage of complete response (CR) or partial response (PR). The disease control rate (DCR) was defined as the proportion of patients with CR or PR or stable disease (SD). Progression-free survival (PFS) was defined as the time from the first dose of SCT200 to the date of PD or death of any reason. OS was defined as the time from the first dose of SCT200 to the date of death due to any reason or last follow-up.

Single-dose PK blood samples were collected within 60 min before dosing and at 0.5, 2, 6, 24, 48, 96, 168, 264, 336, 408, and 504 h after dosing. For multiple dosing, PK blood samples were collected within 60 min before each dose and 30 min after dosing. Intensive blood collection was also performed for the first and last doses.

### Statistical analyses

All statistical analyses were performed using SAS (version 9.4; SAS Institute, Cary, NC, USA). Safety and efficacy evaluations were performed on all patients who received ≥ one dose of SCT200 treatment, and all AEs were recorded according to MedDRA (version 22.1). Survival was estimated using the Kaplan–Meier method, and the time to the onset of AEs of dermotoxicity and hypomagnesemia was analyzed. 95% confidence intervals (CIs) were calculated using the Clopper–Pearson method. Blood concentrations of SCT200 were measured at various time points using an enzyme-linked immunosorbent assay, and key PK parameters were calculated using a non-compartmental model (WinNonlin pharmacokinetic analysis software).

## Results

### Patient disposition and demographics

A total of 56 patients were enrolled from December 16, 2014 to December 31, 2018. All patients were *RAS* (*KRAS*/*NRAS* 2, 3 and 4 exons) and *BRAF V600E* wild-type. 12.5% (7/56) of the patients had right-sided primary colon cancer and 87.5% (49/56) had left-sided primary colon cancer. 96.4% (53/56) of the patients failed at least two prior lines of chemotherapy. Fifty-five patients received multiple doses of SCT200, including 21 patients in the low-dose groups (0.5 mg/kg, 1.0 mg/kg, 2.0 mg/kg, 4.0 mg/kg, 6.0 mg/kg, and 8.0 mg/kg), 9 patients in the high dose groups (8.0 mg/kg, 12.0 mg/kg, and 15.0 mg/kg) and 25 patients in the dose-expansion group (6.0 mg/kg). One patient in the 4.0 mg/kg group received a single dose of SCT200 and did not receive subsequent multiple doses as the patient received other anticancer therapy. After completing the pre-specified multiple-dose treatment cycles, 38 patients continued the treatment with the same treatment regimens. Reasons for terminating SCT200 treatment included PD (*n* = 29), voluntary withdrawal (*n* = 4), and withdrawal after SCT200 treatment was delayed by 2 weeks (*n* = 2). Three patients continued treatment until the data-cutoff date on December 31, 2018 (Fig. 1, Table 1).

**Table 1:** Patient baseline characteristics

Parameter/statistic	Dose-escalation phase (*n* = 31)	Dose-expansion phase (*n* = 25)	Overall (*n* = 56)
Age (years)			
Median (range)	53 (38–70)	56 (30–65)	55 (30–70)
Sex, n (%)			
Male	23 (74.2)	17 (68.0)	40 (71.4)
Female	8 (25.8)	8 (32.0)	16 (28.6)
ECOG PS, n (%)			
0	0 (0)	0 (0)	0 (0)
1	31 (100)	25 (100)	56 (100)
Primary site, n (%)			
Right-sided	5 (16.1)	2 (8.0)	7 (12.5)
Left-sided	26 (83.2)	23 (92.0)	49 (87.5)
Presence of liver metastases, n (%)			
Yes	21 (67.7)	22 (88.0)	43 (76.8)
No	10 (32.3)	3 (12.0)	13 (23.2)
Previous anticancer treatment, n (%)			
Surgery	25 (80.6)	22 (88.0)	49 (87.5)
Radiotherapy	8 (25.8)	11 (44.0)	19 (33.9)
Number of prior chemotherapy lines, n (%)			
1	1 (3.2)	1 (4.0)	2 (3.6)
2	16 (51.6)	9 (36.0)	25 (44.6)
≥ 3	14 (45.2)	15 (60.0)	29 (51.8)
Prior treatment with bevacizumab, n (%)	8 (25.8)	6 (24.0)	14 (25.0)
Disease course (months)			
Median (range)	21.3 (4.4–169.3)	23 (6.2–88.0)	22.2 (4.4–169.3)

Disease course (months) = (time from the date of diagnosis of metastatic colorectal cancer to the date of signing informed consent form + 1) / 30.5

*ECOG* Eastern Cooperative Oncology Group, *PS* Performance status

### Safety

7.1% (4/56) of the patients developed Grade 1–2 infusion reactions at the time of the first dose of SCT200, and no Grade ≥ 3 infusion reactions occurred. No patients reported DLT during the single and multiple dose escalation period. SCT200 dose was not escalated beyond 15.0 mg/kg, and the maximum tolerated dose (MTD) was not reached in this study. All of the patients experienced treatment-emergent adverse events (TEAEs). 96.4% (54/56) of the patients experienced treatment-related adverse events (TRAEs). The most common TRAEs of the 56 patients were dermotoxic-related AEs, including dermotoxicity with acneiform dermatitis (89.3%), paronychia (33.9%), and dry skin (21.4%). Other TRAEs included hypomagnesemia (71.4%), elevated alanine aminotransferase (ALT) (28.6%), elevated aspartate aminotransferase (AST) (17.9%), elevated bilirubin (17.9%), elevated alkaline phosphatase (10.7%), hypertriglyceridemia (25.0%), hypophosphatemia (17.9%), conjunctivitis (16.1%), and hypercholesterolemia (14.3%). Fifteen of 56 patients (26.8%) developed Grade ≥ 3 TRAEs: acneiform dermatitis (8.9%), dry skin (3.6%), and hypomagnesemia (8.9%). Serious adverse events (SAEs) (*n* = 4) included acute myeloid leukemia, gastrointestinal bleeding, intestinal obstruction, and ureteral calculi (one each), all of which were considered unrelated to SCT200 (Supplementary Table 1). The median time to the first dermotoxicity and hypomagnesemia event was 8.0 (95% CI: 6.0–9.0) and 66.0 (95% CI: 41.0–78.0) days, respectively (Supplementary Table 2).

### Pharmacokinetics

**Fig. 2:**
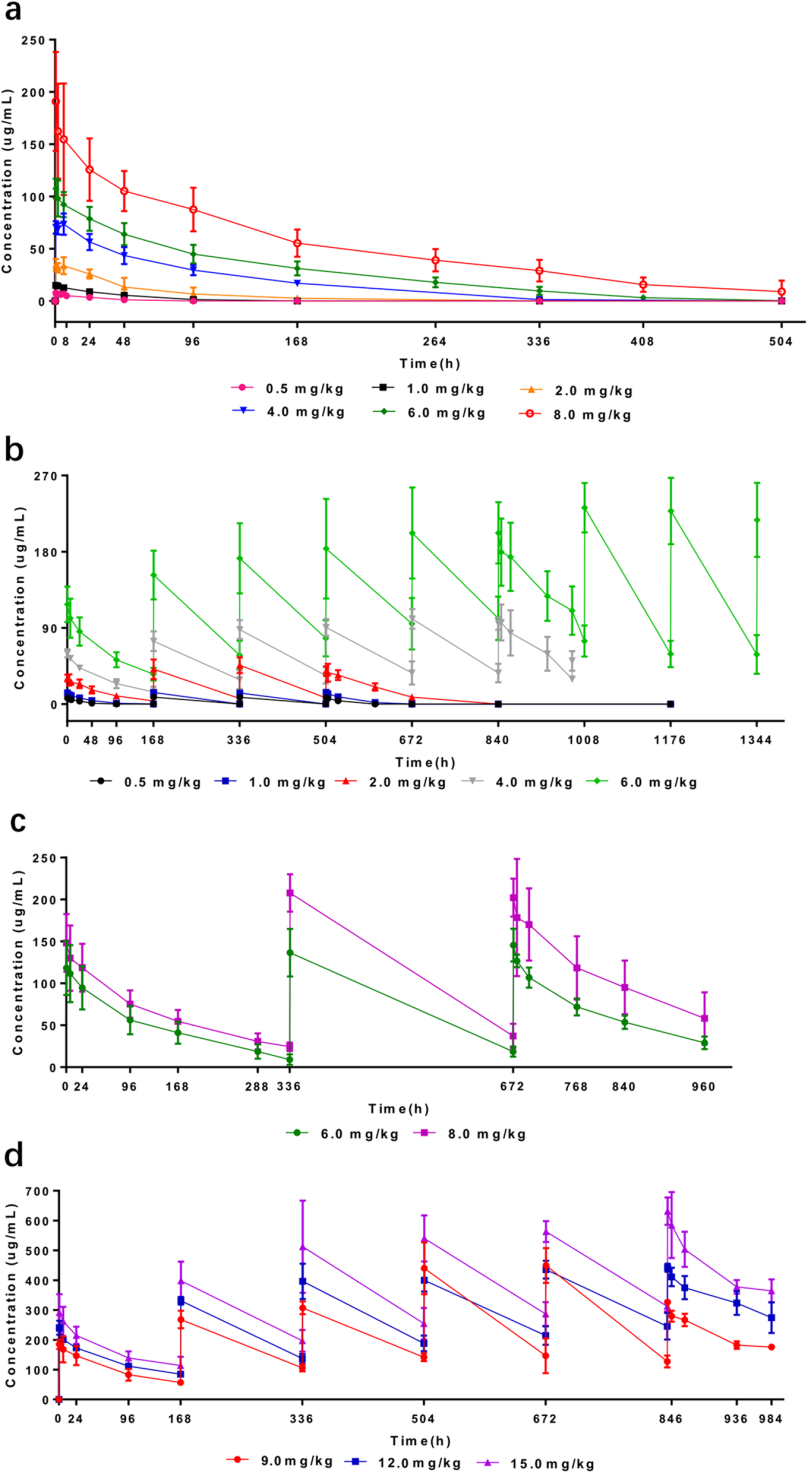
Mean (standard deviation) blood drug concentration–time curves for each dose group of SCT200. Mean (standard deviation) blood drug concentration–time curves for **a** single-dose groups of SCT200 of 0.5 mg/kg (*n*=3), 1.0 mg/kg (*n*=3), 2.0 mg/kg (*n*=3), 4.0 mg/kg (*n*=4), 6.0 mg/kg (*n*=6), and 8.0 mg/kg (*n*=3); **b** multiple-dose groups of SCT200  of 0.5 mg/kg QW (*n*=3), 1.0 mg/kg QW (*n*=3), 2.0 mg/kg (*n*=3), 4.0 mg/kg QW (*n*=3), and 6.0 mg/kg QW (*n*=27); **c** multiple-dose groups of SCT200 of 6.0 mg/kg Q2W (*n*=3), and 8.0 mg/kg Q2W (*n*=3); and **d** multiple-dose groups of SCT200 of 9.0 mg/kg QW (*n*=3), 12.0 mg/kg QW (*n*=3), and 15.0 mg/kg QW (*n*=3). QW Quaque week, Q2W Quaque 2 week

The SCT200 single-dose PK investigation was performed at six dose levels (0.5 mg/kg, 1.0 mg/kg, 2.0 mg/kg, 4.0 mg/kg, 6.0 mg/kg, and 8.0 mg/kg). The concentration–time curves of different doses of SCT200 are shown in Fig. 2a and the major PK parameters are presented in Table 2. The results showed that, as the dose of SCT200 increased from 0.5 mg/kg to 8.0 mg/kg, the maximum plasma concentration (C_max_) appeared to be approximately dose proportional, the area under the concentration–time curve (AUC) exposure disproportionally increased, clearance decreased from 2.3 mL/h/kg to approximately 0.3 mL/h/kg, and the elimination half-life was extended from 17.6 h to 75.9 h.

**Table 2:** PK parameters after a single dose of SCT200

Dose (mg/kg)	n	C_max_ (μg/mL)	AUC_0-∞_ (h*μg/mL)	CL_obs (mL/h/kg)	T_1/2_ (h)	T_max_ (h)^a^	Vz_obs (ml/kg)
0.5	3	7.7 (13.2)	216.7 (17.7)	2.3 (17.7)	17.6 (7.6)	2.5 (2.5–2.5)	58.7 (10.8)
1.0	3	15.8 (11.4)	665.7 (12.7)	1.5 (12.7)	20.0 (4.8)	2.7 (2.5–4.0)	43.3 (16.1)
2.0	3	36.7 (20.4)	2,048.8 (44.2)	1.0 (44.2)	36.7 (57.7)	2.5 (2.5–7.6)	51.7 (22.1)
4.0	4	74.6 (11.9)	8,037.3 (15.9)	0.5 (15.9)	52.9 (40.0)	5.2 (2.5–8.0)	37.9 (31.8)
6.0	6	109.0 (10.0)	13,176.4 (19.9)	0.5 (19.9)	67.2 (37.8)	2.5 (2.5–4.0)	44.1 (42.1)
8.0	3	186.7 (26.9)	27,216.0 (31.2)	0.3 (31.2)	75.9 (115.8)	2.5 (2.5–2.5)	32.2 (75.4)

The results shown are geometric means (coefficient of variation of the geometric mean)

*AUC*_0-∞_ area under the concentration–time curve from time zero to infinity, *C*_max_ maximum plasma concentration, *CL* Clearance, *T*_1/2_ half-life, *T*_max_ time to maximum plasma concentration, *Vz* volume of distribution, *PK* pharmacokinetic

^a ^T_max_ is shown as median (range)

The drug concentration–time curves for multiple doses of SCT200 are shown in Fig. 2b-d and the major PK parameters are presented in Table 3. The area under the concentration–time curve over a dosing interval (AUC_tau_) increased following the dose ascending from 0.5 mg/kg to 15.0 mg/kg. The AUC_tau_ increased disproportionally when the dose escalated from the 0.5 mg/kg to 4.0 mg/kg, showing a non-linear PK characteristic. When the dose escalated from 6.0 mg/kg to 15.0 mg/kg, the AUC_tau_ approximately increased proportionally, showing a linear PK trend. The accumulation index fluctuated in a range from 1.3 to 2.4 when the dose escalated from 0.5 mg/kg to 15.0 mg/kg. Following the prolonged multi-dose treatment of SCT200, all groups showed a decrease in the drug clearance, increase in the peak plasma concentration, and a prolonged T_1/2_. Based on the PK results of SCT200 dose escalation and comparison of similar drugs, 6.0 mg/kg was determined as the target dose of SCT200 for the dose-expansion phase, and a steady state was reached after five consecutive SCT200 administrations.

**Table 3:** PK parameters for multiple dosing of SCT200

Dose group	0.5 mg/kg QW	1.0 mg/kg QW	2.0 mg/kg QW	4.0 mg/kg QW	6.0 mg/kg QW	6.0 mg/kg Q2W	8.0 mg/kg Q2W	9.0 mg/kg QW	12.0 mg/kg QW	15.0 mg/kg QW
n	*n* = 3	*n* = 3	*n* = 3	*n* = 3	*n* = 17	*n* = 3	*n* = 3	*n* = 3	*n* = 3	*n* = 3
AUC_tau_ (h*µg/mL)	280.0 (13.7)	674.1 (43.8)	3,788.3 (19.9)	10,552.4 (33.0)	23,231.0 (22.0)	19,577.6 (15.3)	32,545.7 (40.9)	35,343.9 (1.9)	55,113.3 (10.0)	70,988.8 (9.5)
AUC_0-∞_ (h*µg/mL)	280.0 (13.8)	675.7 (44.1)	4,479.0 (19.5)	18,080.2 (43.7)	51,260.6 (33.7)	23,931.8 (19.7)	46,099.0 (60.2)	80,008.9 (16.1)	154,936.3 (47.1)	192,598.2 (23.8)
Accumulation index	1.6 (7.2)	1.3 (17.9)	1.8 (12.1)	2.0 (24.0)	2.1 (13.8)	1.3 (20.2)	1.6 (31.0)	1.9 (18.4)	2.4 (5.0)	2.4 (5.8)
CL_ss_ (mL/h/kg)	1.8 (13.8)	1.5 (43.8)	0.5 (19.9)	0.4 (33.0)	0.3 (22.0)	0.3 (15.3)	0.25 (40.9)	0.3 (1.9)	0.2 (10.0)	0.2 (9.5)
C_max_ (µg/mL)	7.7 (8.4)	13.9 (16.6)	39.5 (17.8)	100.0 (16.6)	196.4 (18.0)	144.8 (13.1)	204.4 (12.0)	326.2 (2.3)	442.0 (3.1)	652.1 (4.9)
C_min_ (µg/mL)	0.1 (56.7)	0.1 (80.0)	7.4 (26.2)	35.6 (32.4)	93.2 (18.1)	18.0 (35.8)	35.0 (48.9)	126.3 (15.9)	241.1 (18.5)	311.9 (5.5)
T_1/2_ (h)	12.7 (9.2)	18.0 (42.1)	28.2 (3.2)	134.2 (13.6)	187.9 (26.6)	138.0 (12.2)	188.7 (41.2)	190.1 (24.5)	263.4 (51.9)	244.3 (32.9)

*AUC*_0-∞_ Area under the concentration–time curve from time zero to infinity, *AUC*_*tau*_ Area under the concentration–time curve over a dosing interval, *C*_max_ Maximum plasma concentration, *C*_min_ Minimum plasma concentration, *CL* Clearance, *QW* Quaque week, *Q2W* Quaque 2 week, *T1/2* Half-life, *PK* Pharmacokinetic

### Efficacy

**Fig. 3:**
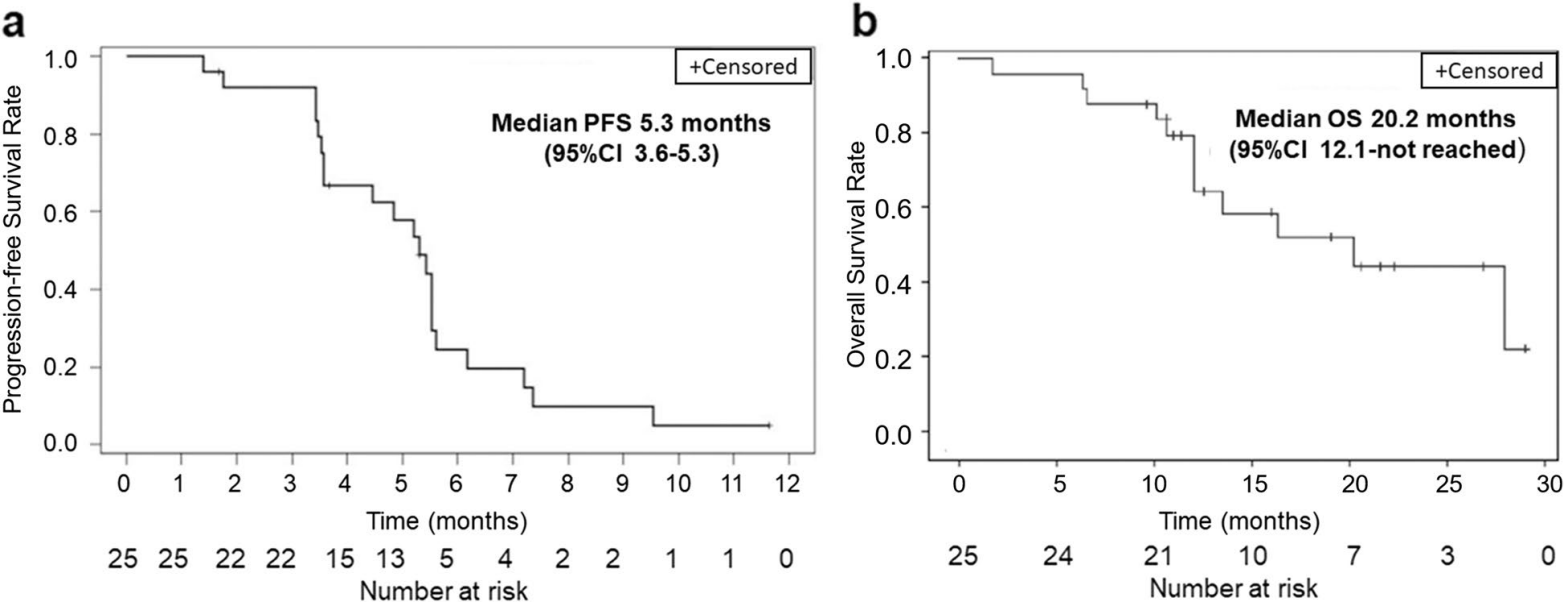
Progression-free survival (PFS) (**a**) and overall survival (OS) (**b**) in the 6.0 mg/kg QW dose-expansion group. CI Confidence interval, QW Quaque week

Efficacy evaluation was performed for all patients who received at least one dose of SCT200. Of 56 patients, 17 achieved PR, with an ORR of 30.4% (95% CI: 18.8%–44.1%), and a DCR of 69.6% (95% CI: 55.9%–81.2%). No patients in the ≤ 4 mg/kg dose groups achieved an objective response. Table 4 summarized the efficacy of SCT200 at all doses. For the dose-expansion group that received a target dose of 6.0 mg/kg QW, 48.0% (12/25) of the patients achieved PR, the ORR was 48.0% (95% CI: 27.8%–68.7%), and the DCR was 92.0% (95% CI: 74.0%–99.0%); the median PFS was 5.3 months (95%CI: 3.6–5.5), and the median OS was 20.2 months (95% CI: 12.1–not reached) (Fig. 3). The presence and severity of skin toxicities were associated with improved clinical efficacy in patients receiving SCT200. Among 56 patients treated with SCT200, the ORRs and median PFS were 16.7% (95% CI: 0.4%-64.1%) and 2.6 months (95%CI: 2.3-not reached) for patients with no evidence of dermotoxicity (*N* = 6), 39.0% (95% CI: 24.2%-55.5%) and 4.4 months (95%CI:3.4–5.5) for patients with Grade 1–2 dermotoxicity (*N* = 41) and 55.6% (95% CI: 21.2%-86.3%) and 5 months (95%CI: 5.2-not reached) for the patients with Grade 3–4 dermotoxicity (*N* = 9), respectively.

**Table 4:** Efficacy of SCT200

Dose	0.5 mg/kg QW	1.0 mg/kg QW	2.0 mg/kg QW	4.0 mg/kg QW	6.0 mg/kg QW	6.0 mg/kg Q2W	8.0 mg/kg Q2W	9.0 mg/kg QW	12.0 mg/kg QW	15.0 mg/kg QW	Overall
	(*N*=3)	(*N*=3)	(*N*=3)	(*N*=4)	(*n*=28)	(*n*=3)	(*n*=3)	(*n*=3)	(*n*=3)	(*n*=3)	(*n*=56)
Efficacy, n											
CR	0	0	0	0	0	0	0	0	0	0	0
PR	0	0	0	0	12	2	0	0	2	1	17
SD	0	0	1	2	12	1	2	2	1	1	22
PD	3	2	2	2	3	0	1	1	0	1	15
NE	0	1	0	0	1	0	0	0	0	0	2
ORR (CR/PR), % (95% CI)	0 (0.0–70.8)	0 (0.0–70.8)	0 (0.0–70.8)	0 (0.0–60.2)	42.9 (24.5–62.8)	66.7 (9.4–99.2)	0.0 (0.0–70.8)	0.0 (0.0–70.8)	66.7 (9.4–99.2)	33.3 (0.8–90.6)	30.4 (18.8–44.1)
DCR (CR/PR/SD), % (95% CI)	0 (0.0–70.8)	0 (0.0–70.8)	33.3 (0.8–90.6)	50 (6.8– 93.2)	85.7 (67.3–96.0)	100.0 (29.2–100.0)	66.7 (9.4–99.2)	66.7 (9.4–99.2)	100.0 (29.2–100.0)	66.7 (9.4–99.2)	69.6 (55.9–81.2)

*CI* Confidence interval, *CR* Complete response, *PR* Partial response, *SD* Stable disease, *PD* Progressive disease, *NE* Non-evaluable, *QW* Quaque week, *Q2W* Quaque 2 week, *ORR* Objective response rate, *DCR* Disease control rate

## Discussion

CRC is the third most common cancer worldwide, with 20–25% of patients initially diagnosed with stage IV disease. The 5-year OS rate of stage IV CRC was 12% [8, 9]. Over the last decade, anti-EGFR monoclonal antibodies have been established as essential drugs for the treatment of wild-type *KRAS/NRAS/BRAF* mCRC. In patients with wild-type *KRAS* mCRC who have failed standard chemotherapy, anti-EGFR monoclonal antibody therapy provided a median OS of 8.1–10.4 months, a median PFS of 2.9–4.4 months, and an ORR of 12.8–27% [10–13].

In this study, SCT200 is well tolerated with no DLT occurring during the dose-escalation phase and no serious TRAEs were observed. The MTD was not reached when the dose of SCT200 escalated to 15.0 mg/kg. The most common TRAE was dermotoxicity. The overall incidence and incidence of Grade ≥ 3 TRAEs of dermotoxicity were 89.3% and 16.1%, respectively, which are comparable to the incidence rates of cetuximab (88% and 10%) and panitumumab (86% and 12%) [13]. Patients in this study generally tolerated SCT200, with only one patient discontinuing the treatment due to dermotoxicity and > 90% recovering with symptomatic treatment or did not require treatment. The median time to the onset of dermotoxicity was 8 days with SCT200, which was similar to that of cetuximab (14 days) and panitumumab (7 days) [14, 15]. A higher incidence of hypomagnesemia was observed on this study than the incidence reported from cetuximab and panitumumab, although most AEs were Grade 1–2. Grade 1–2 infusion reaction was experienced by 7.1% of patients, and no Grade ≥ 3 infusion reactions occurred. In contrast, cetuximab has the incidence of infusion-related AEs of 8.4%, and 2.2% were Grade ≥ 3 [15].

The target dose of SCT200 was determined based on the PK investigation of dose-escalation phase. SCT200 was well tolerable within the dose level of 15.0 mg/kg. The target steady-state trough concentrations of cetuximab and panitumumab were 41–85 μg/mL and 50 μg/mL, respectively [14]. Considering that SCT200 had lower in vivo exposure than the same dose of panitumumab, a steady-state trough concentration comparable to cetuximab and panitumumab could be achieved at 4.0 mg/kg to 6.0 mg/kg quaque week (QW) for six consecutive doses, or at a dose frequency of 8.0 mg/kg quaque 2 week (Q2W) for three consecutive doses of SCT200.

In this study, 6.0 mg/kg QW was determined as the target dose for the dose-expansion study. 96.0% (24/25) who failed at least two prior lines of chemotherapy, were enrolled to receive 6.0 mg/kg QW for six consecutive doses and then changed to 8.0 mg/kg Q2W to improve patient compliance. 48.0% (12/25, 95% CI: 27.8%–68.7%) of the patients had PR. The DCR was 92.0% (23/25, 95% CI: 74.0%–99.0%), the median PFS was 5.3 months (3.6–5.5), and the median OS was 20.2 months (95% CI: 12.1–not reached). In the phase III study of panitumumab *versus* best supportive care [12], the ORR, median PFS, and median OS in patients with wild-type *RAS* (*KRAS* and *NRAS* exons 2, 3, and 4) mCRC in the panitumumab group was 31%, 5.2 months, and 10  months, respectively. The study result of cetuximab showed that the ORR was 12.8%, median PFS was 3.7 months, and median OS was 9.5 months in patients with wild-type *KRAS* exon 2 mCRC [13]. Previous study showed that patients with left side colon cancer had significantly improved PFS when treated with cetuximab compared to those with right side colon cancer [16]. This study had higher proportion of patients with left side colon cancer (87.5%, 49/56) in comparison with those in studies of panitumumab (42%) and cetuximab (35%), and this may contribute to the higher ORR and longer OS [13]. This study is limited by the small sample size. Larger sample studies are warranted to verify its efficacy and safety profile.

## Conclusions

This phase I dose-escalation and dose-expansion study demonstrated that SCT200 showed a favorable safety, tolerabolity, PK profile, and efficacy to patients with wild-type *KRAS/NRAS/BRAF* mCRC.

## Supplementary Information

**Additional file 1.** Protocol synopsis.

**Additional file 2:** **Supplementary Table 1.** Occurrence of adverse events associated with study drugs (overall incidence >10%).

**Additional file 3:** **Supplementary Table 2.** Kaplan–Meier method—time to first dermotoxicity/hypomagnesemia event.

## Data Availability

The datasets used and/or analyzed during the current study are available from the corresponding author on reasonable request.

## Abbreviations

AUC: Area under the concentration–time curve
AUC_tau_: Area under the concentration–time curve over a dosing interval
CI: Confidence interval
CR: Complete response
CRC: Colorectal cancer
CTCAE: Common Terminology Criteria for Adverse Events
DLT: Dose-limiting toxicity
ECOG: Eastern Cooperative Oncology Group
PS: Performance status
EGFR: Epidermal growth factor receptor
MAD: Multiple ascending dose
MTD: Maximum tolerated dose
ORR: Objective response rate
OS: Overall survival
PFS: Progression-free survival
PK: Pharmacokinetic
PR: Partial response
RECIST: Response Evaluation Criteria in Solid Tumors
SAD: Single ascending dose
SAE: Serious adverse event
SD: Stable disease
TRAE: Treatment-related adverse events
TEAE: Treatment-emergent adverse events
QW: Quaque week
Q2W: Quaque 2 week
