# Supplementary material for: Safety, pharmacokinetics and efficacy of SCT200, an anti-EGFR monoclonal antibody in patients with wild-type KRAS/NRAS/BRAF metastatic colorectal cancer: a phase I dose-escalation and dose-expansion study
Source: BMC Cancer. 2022 Oct 28;22:1104. doi: 10.1186/s12885-022-10147-9 (PMC9617324; doi:10.1186/s12885-022-10147-9)
Supplement: Supplementary file 1 — Additional file 1. Protocol synopsis. [file 12885_2022_10147_MOESM1_ESM.docx]

**Protocol Synopsis**

**Study title**:

A non-randomized, open, single and multiple dose escalation Phase I clinical trial to evaluate the safety, tolerability and pharmacokinetics of recombinant fully human anti-human epidermal growth factor receptor (EGFR) monoclonal antibody injection (SCT200) in patients with metastatic colorectal cancer who have failed treatment with fluorouracil, oxaliplatin and irinotecan.

**Objective**:

The primary objectives of this study are to evaluate the safety and tolerability and to assess the pharmacokinetics of SCT200 intravenous single and multiple doses in patients with metastatic colorectal cancer who have failed treatment with fluorouracil, oxaliplatin and irinotecan.

The secondary objective is to initially evaluate the clinical efficacy of SCT200 and to provide a basis for dose selection for the Phase II clinical trial of this product.

**Study design**:

This study is a single-center, non-randomized, open, dose-escalating Phase I clinical trial. The trial is set up as a single dose and a multiple dose. Subjects who complete the single dose safety and tolerability assessment can continue into the multiple dose trial at the original dose level after a 3-week washout period.

Safety, tolerability and pharmacokinetic assessments of multiple dosing were completed by the protocol deadline and this study was concluded. Subjects who complete multiple dosing with tumor assessment in remission or stable disease may continue to follow-up maintenance therapy until disease progression or intolerable toxicity occurs.

The revisions to each version of the protocol that occurred during the course of the trial are detailed in Table 1 below (the study started with version 2.0 of the protocol).

**Table 1. Summary of revised information for each version of the program**

| Version change | Main changes |
| --- | --- |
| Original version：2.0/2014-07-15  New version：2.1/2015-01-22 | Update blood collection point settings: reduce 2 blood collection points and add a new blood collection point |
| Original version：2.1/2015-01-22  New version：3.0/2015-11-10 | 1. Revised climbing dose group: 3.0mg/kg and 5.0mg/kg dose groups were eliminated and the highest dose was set at 8.0mg/kg and study period； 2. Revision: Immunogenicity and pharmacogenetic blood collection points； |
| Original version：3.0/2015-11-10  New version：3.1/2016-04-01 | Increased maintenance component with bimonthly immunogenicity assessments |
| Original version：3.1/2016-04-01  New version：3.2/2016-06-12 | new blood collection points for drug substitutes in the single dose section； |
| Original version：3.2/2016-06-12  New version：3.3/2017-07-31 | Added ctDNA non-invasive individualized diagnosis and treatment genetic testing content as an exploratory project for efficacy prognosis and other studies |
| Original version：3.3/2017-07-31  New version：4.0/2017-09-15 | A total of 3 dose escalation groups of 9.0mg/kg/week, 12.0mg/kg/week and 15.0mg/kg/week were added, and the new dose groups were not subjected to single dose pharmacokinetic studies. |
| Original version：4.0/2017-09-15  New version：5.0/2017-12-15 | The dosing regimen for the expanded entry group changed to SCT200 6.0 mg/kg given once a week for 6 weeks, followed by 8.0 mg/kg given once every 2 weeks for continuous dosing. |

1. **Version 2.1 protocol revision**

The version 2.1 protocol has seven dose components of 0.5 mg/kg, 1.0 mg/kg, 2.0 mg/kg, 3.0 mg/kg, 4.0 mg/kg, 5.0 mg/kg and 6.0 mg/kg. The single dose portion of the dose was administered group by group starting with the first dose of 0.5 mg/kg according to the dose creep principle (see version 2.1 protocol section 4.4.6). Subjects may continue into the multiple dose portion at the original dose level after a 3-week washout period following completion of the single dose safety and tolerability assessment. The starting dose for the sequential multiple dosing trial will also be 0.5 mg/kg, and each dose group will be cascaded up to a maximum of 6.0 mg/kg, or to the maximum tolerated dose (MTD) of the single dosing trial, as in the single dosing trial. The dose regimen for the 4.0 mg/kg dose group will be based on the results of the single dose pharmacokinetic analysis, as well as the results of the sequential multiple dose pharmacokinetic analysis for the preceding dose groups, and will be determined by either a four-dose per week (QW, 4 dose) or two-dose per two-week (Q2W, 2 dose) intravenous dosing regimen. The single and multiple dosing regimens and the design of dose group levels and escalation, in particular the lowest dose of 0.5 mg/kg and the highest dose of 6.0 mg/kg (see protocol sections 4.4.3 and 4.4.4 of version 2.1), are based on the results of preclinical studies of SCT200, in addition to preclinical, clinical studies and the dosing regimen of the same target drug panitumumab.

The safety and tolerability studies and pharmacokinetic studies have been completed for three dose groups: 0.5 mg/kg, 1.0 mg/kg and 2.0 mg/kg. The results showed that the blood concentration of SCT200 at 2.0 mg/kg was equivalent to that of panitumumab at 1.5 mg/kg, and the skin toxicity was also milder than that of panitumumab at the same dose level, suggesting that the exposure of SCT200 may be lower than that of panitumumab. The results of panitumumab showed that EGFR-mediated systemic clearance saturated at a dose level of 2.5 mg/kg/week, so the dose level of 2.5 mg/kg/week was confirmed as the optimal clinical dose; currently, the recommended clinical dosing regimen for panitumumab is 6.0 mg/kg once every two weeks, alone or in combination with chemotherapy. Based on the results of the completed SCT200 dose group safety and pharmacokinetic study, it was decided to modify the subsequent single and multiple dose group increments, maximum dose group settings, and consecutive multiple dosing cycles on the basis of controlled risk and maximum protection of subjects' rights and interests.

1. **Version 3.0 protocol revision**

Version 3.0 protocol revisions were made with reference to the results of the prior trials of this clinical study, as well as input from the principal investigator and pharmacogenetic experts. The main considerations and basis for revision are summarized below.

Given the current pre-trial results of SCT200, which showed pharmacokinetic parameters of 2.0 mg/kg and skin toxicity equivalent to the 1.5 mg/kg level of panitumumab, it is speculated that the optimal weekly clinical dose of SCT200 may be 4.0 mg/kg, and the recommended clinical dose for biweekly dosing may be 8.0 mg/kg. Therefore, the maximum dose was adjusted to 8.0 mg/kg.

Also, because only minimal toxicity and efficacy were observed in the 2.0 mg/kg dose group, the dose escalation was adjusted in version 3.0 to 2.0 mg/kg directly to 4.0 mg/kg to 6.0 mg/kg (deleting the 3.0 mg/kg and 5.0 mg/kg dose groups from the original version 2.1) to a maximum dose of 8.0 mg/kg in order to avoid more subjects using ineffective drugs. In addition to the accelerated dose escalation, the 3.0 regimen also revised the dosing intervals for the last three dose groups from 4.0 mg/kg for 4 weeks in the 2.1 version to 6 weeks in the 3.0 version, i.e., the 4.0 mg/kg dose group received six consecutive doses once a week; the 8.0 mg/kg dose group will receive three consecutive doses every two weeks; the 6.0 mg/kg dose group will receive either six consecutive weekly doses (QW, 6dose) or three consecutive doses every two weeks (Q2W, 3dose) based on the results of the pharmacokinetic analysis of the single dose and the pharmacokinetic results of the consecutive doses in the 4.0 mg/kg dose group.

In order to more accurately analyze the pharmacokinetic characteristics of SCT200, based on the recently updated pharmacokinetic results at 4.0 mg/kg, this version 3.2 protocol will add two additional pharmacokinetic blood collection points in the single dose section, and decide to add blood collection at 11 and 17 days after single dose in the 6.0 mg/kg and later dose groups, as detailed in the flow chart.

This version will be described according to six dose groups: 0.5 mg/kg, 1.0 mg/kg, 2.0 mg/kg, 4.0 mg/kg, 6.0 mg/kg, and 8.0 mg/kg. In view of the observation and evaluation of the three dose groups of 0.5mg/kg, 1.0mg/kg and 2.0mg/kg, the 3.0 version of the protocol focuses on the safety evaluation and pharmacokinetic studies of 4.0mg/kg, 6.0mg/kg and 8.0mg/kg, and the multiple dosing section is mentioned for the six-week dosing of these three dose groups. The first three dose groups of four weeks of dosing are not mentioned in the study protocol version 2.1. To avoid excessive revisions, only the inconsistencies with the original protocol were added in version 3.0.

1. **Version 3.3 protocol revision**

Version 3.3 of the protocol was modified by referring to similar target drugs, new directions in colorectal cancer treatment research, and consulting with principal investigators. The revision added ctDNA non-invasive individualized diagnosis and treatment genetic testing as an exploratory project to conduct research on efficacy prognosis and other studies.

Patients will undergo genetic testing of their first blood sample at baseline and a second blood sample genetic test after continuous dosing at 6.0 mg/kg/week (i.e., at early withdrawal/end-of-study visit). Patients enter follow-up maintenance therapy and will undergo genetic testing of blood samples every 2 months. See flow chart for detailed process - [appendix II](#_附件一：单次给药试验流程表) section.

Changes to PK blood collection points for multiple consecutive doses: The revised protocol will only retain PK blood collection points within 60 minutes prior to and 30 minutes ± 3 minutes after the end of each dose for doses 1-6 (consecutive multiple dosing periods), i.e., eliminating: the original blood collection points at 6 hours, 24 hours, and 4 days after the first intravenous dose; and the original blood collection points at 6 hours, 24 hours, 4 days, and 6 days after the last intravenous dose. Refer to the flow chart for specific implementation process - appendix II section.

Except for the changes listed above, no other changes were made compared to the implemented version 3.2 of the protocol

1. **Version 4.0 protocol revision**

Prior to the revision of version 4.0 of the protocol, the target dose was determined to be 6.0 mg/kg/week based on pharmacogenetic results through dose-escalation exploration and observation in seven dose groups of 0.5 mg/kg/week, 1.0 mg/kg/week, 2.0 mg/kg/week, 4.0 mg/kg/week, 6.0 mg/kg/2 weeks, 6.0 mg/kg/week and 8.0 mg/kg/2 weeks. The target dose was 6.0 mg/kg/week, followed by an expansion study in the 6.0 mg/kg/week dose group (since version 5.0 of the protocol, the expanded enrollment dosing regimen was revised to: SCT200 6.0 mg/kg administered once weekly for 6 weeks, followed by 8.0 mg/kg administered once every 2 weeks for continuous dosing treatment).

Given that no DLT was seen in the dose-escalation study, and that data from the current expanded enrollment study showed that 6.0 mg/kg/week was safe and well tolerated and also demonstrated good efficacy, the dose-escalation study is being continued with a revised protocol to version 4.0.

Compared with version 3.3 of the protocol, version 4.0 adds three additional dose escalation groups (new dose groups) at 9.0 mg/kg/week, 12.0 mg/kg/week, and 15.0 mg/kg/week, and no single dose pharmacokinetic study is conducted in the new dose groups. At the additional doses of 9.0 mg/kg/week, 12.0 mg/kg/week, and 15.0 mg/kg/week, patients were enrolled in dose escalation from low to high doses according to the dose escalation principle (see subsection 4.4.6 of the text for the dose escalation principle), and received continuous dosing of the study drug while undergoing multiple dosing pharmacokinetic analysis. Three to six subjects were planned to be enrolled in each dose group.

Pharmacogenetic blood collection points were set: within 60 minutes before the first intravenous administration, 30±3 minutes, 6 hours, 24 hours and 4 days after the end of intravenous administration; within 60 minutes before and 30 minutes after the end of the second, third, fourth and fifth administration; and once each within 60 minutes before and 30 minutes, 6 hours, 24 hours, 4 days and 6 days after the end of the last intravenous administration.

**Enrolled principles:**

During the study, patients were prioritized into the new dose group, and after the current dose group enrollment was completed and before the next dose group began, newly screened patients could enter the expanded enrollment study at 6 mg/kg/week.

Beginning with version 5.0, the expanded entry dosing regimen was revised to include SCT200 6.0 mg/kg administered weekly for 6 weeks, followed by 8.0 mg/kg administered every 2 weeks for continuous dosing. Subjects entering maintenance dosing (those who have completed SCT200 6.0 mg/kg for more than 6 weeks) should follow this revised regimen and subsequently receive 8.0 mg/kg every 2 weeks for continuous dosing.

Patients signed an informed consent form and entered the study screening period. Patient demographic data, past medical history, current medical history, history of antineoplastic therapy, history of previous 30-day medication use, and RAS test results, physical examination, and tumor imaging (including cranial, thoracic, abdominal, and pelvic) were collected within 21 days prior to the first dose. Within 7 days prior to the first dose, previous 30-day medication was collected, physical examination was performed, height, weight, vital signs were measured, physical status score was performed according to ECOG PS, and 12-lead ECG, pregnancy test, and laboratory tests were completed. Patient eligibility was confirmed 1 day before the first dose according to the study's enrollment criteria.

1. **Version 4.0 protocol revision**

Based on the results of the current phase I study, the revised version 5.0 regimen is planned to change the original 6.0 mg/kg/week (continuous multiple dosing and subsequent maintenance treatment) dosing regimen for the expanded enrollment group to a continuous dosing regimen of SCT200 6.0 mg/kg once a week for 6 weeks, followed by 8.0 mg/kg once every 2 weeks. Subjects entering maintenance dosing (those who have completed SCT200 6.0 mg/kg for more than 6 weeks) should follow this revised regimen and subsequently receive 8.0 mg/kg every 2 weeks as a continuous dosing regimen.

Pharmacogenetic blood collection point settings: for the first three subjects enrolled in the study, SCT200 6.0 mg/kg was administered intravenously six consecutive times during the weekly dosing phase, and blood was collected within 60 minutes before and 30 minutes ± 3 minutes after the end of each dose; SCT200 8.0 mg/kg was administered during the 2-weekly dosing phase, and blood was collected within 60 minutes before and 30 minutes ± 3 minutes after the end of each dose. Blood samples were collected only during the first three consecutive intravenous doses. See Flowchart-Appendix II and Appendix IV sections for specific implementation procedures. For subjects who continue to be enrolled (after 3 subjects), no pharmacokinetic study will be performed and the immunogenicity study will remain unchanged, see Flowchart - Appendix II and Appendix IV for specific procedures.

Study treatment endpoints until disease progression or until intolerable toxicity occurs.

**Brief description of the study.**

This study was divided into two parts: single dosing and multiple dosing. Eligible patients are first entered into the first part of the study, the single dose trial, and are divided into six dose groups of 0.5 mg/kg, 1.0 mg/kg, 2.0 mg/kg, 4.0 mg/kg, 6.0 mg/kg, and 8.0 mg/kg, which are administered group by group according to the dose creep principle (see Section 4.4.6). The dose for each patient will be calculated based on their body weight. Safety and tolerability will be observed before, during, and 48 hours, 4 days, and 7 days after intravenous administration, and safety and tolerability data will be collected at 7-day follow-up until 21 days after intravenous administration. Laboratory tests were performed every 7 days after intravenous administration until 21 days after intravenous administration. Cardiac monitoring was performed before, during, and 1 hour after infusion, and the subject's vital signs (temperature, respiration, blood pressure, and heart rate) were closely monitored, as well as facial color and the presence of sweating or headache, for early detection of signs of infusion reactions. An electrocardiogram was performed 21 days after routine intravenous administration, or at any time during the study if deemed necessary by the investigator. Immunogenic blood samples were collected within 60 minutes prior to the start of intravenous administration and 21 days after intravenous administration.

Pharmacokinetic blood samples were collected within 60 minutes prior to intravenous administration, 30 minutes, 2 hours, 6 hours, 24 hours, 48 hours, 4 days, 7 days, 11 days, 14 days, 17 days, and 21 days after the end of intravenous administration.

The second part is a multiple dosing trial. Subjects who have completed the single dose tolerance and safety assessment may continue into the multiple dosing trial at the original dose level after a 3-week washout period. The starting dose for the sequential multiple dosing trial is 0.5 mg/kg, and each dose group will be cascaded up to a maximum of 8.0 mg/kg, or to the maximum tolerated dose (MTD) of the single dose trial, as in the single dose trial. The 0.5 mg/kg dose group to the 2.0 mg/kg dose group received four consecutive weekly doses, the 4.0 mg/kg dose group received six consecutive weekly doses, and the 8.0 mg/kg dose group received three consecutive intravenous doses every two weeks. The 6.0 mg/kg dose group will receive either six consecutive weekly doses (QW, 6 dose) or/and three consecutive doses every two weeks (Q2W, 3dose)based on the results of the single dose pharmacokinetic analysis, as well as the results of the consecutive dose pharmacokinetics of the previous dose groups. Tolerability and safety data were observed at weekly follow-up visits during the treatment period of administration for each dose group. Cardiac monitoring was performed before, during, and 1 hour after infusion, and the subjects' vital signs (temperature, respiration, blood pressure, and heart rate) and facial color, sweating, or headache were closely monitored for early detection of signs of infusion reactions. Electrocardiograms and tumor assessments were routinely performed after the last dose, and in the investigator's judgment, electrocardiograms could be performed at any time if necessary. Immunogenic blood samples were collected before each dose.

Blood samples for the weekly, 6 consecutive dosing pharmacokinetic study were collected within 60 minutes before and 30 minutes ± 3 minutes after the end of the first intravenous dose; within 60 minutes before and 30 minutes ± 3 minutes after the end of the second, third, fourth and fifth doses; and within 60 minutes before and 30 minutes ± 3 minutes after the end of the last intravenous dose. Blood samples for the pharmacokinetic study were collected every 2 weeks for 3 consecutive doses: within 60 minutes before the first intravenous dose, 30 minutes after the end of intravenous dose, 6 hours, 24 hours, 4 days, 7 days and 12 days; within 60 minutes before the second dose and 30 minutes after the end of dose; within 60 minutes before the third dose, 30 minutes after intravenous dose, 6 hours, 24 hours, 4 days, 7 days and 12 days after the third administration.

Patients receive continuous weekly or biweekly dosing, and if possible, patients schedule each dose at the same time.

The additional dose group underwent weekly continuous dosing, and blood samples for the pharmacokinetic study were set at the following times: within 60 minutes before the first intravenous dose, 30 ± 3 minutes after the end of intravenous dosing, 6 hours, 24 hours, and 4 days; within 60 minutes before the second, third, fourth, and fifth doses, and 30 minutes after the end of dosing; and within 60 minutes before the last intravenous dose, 30 minutes after the end of intravenous dosing, and 6 hours, 24 hours, 4 days, and 6 days were collected once each.

After the revision of protocol version 5.0, based on the treatment protocol of SCT200 6.0 mg/kg administered once a week for 6 weeks, followed by 8.0 mg/kg administered once every 2 weeks, the timing of blood sample collection for the pharmacokinetic study was set as follows: during the weekly dosing phase, six consecutive intravenous doses were collected within 60 minutes before and 30 minutes ± 3 minutes after the end of each dose; during the 2-week dosing phase, blood samples were collected within 60 minutes before and 30 minutes ± 3 minutes after the end of each dose, and only the first three consecutive intravenous doses were collected. During the weekly dosing phase, blood was collected within 60 minutes before and 30 minutes ± 3 minutes after each dose, and only the first three consecutive intravenous doses were collected.

End of study: Patients receive multiple doses of treatment for 6 weeks, and the study ends after safety and tolerability assessments and pharmacokinetic studies.

Patients who receive multiple doses and whose tumors are assessed to be in disease remission or stable may be allowed to receive maintenance therapy at their respective dose levels until disease progression occurs or intolerable toxicities develop. After evaluation, patients may be allowed to receive maintenance therapy with repeat dosing at the target dose if the low dose group is not considered to provide the greatest benefit. During follow-up maintenance treatment, if drug-related adverse events occur but the treatment is judged by the investigator to be beneficial to the patient, the dose is allowed to be adjusted downward, to the previous lower dose level, or suspended at the investigator's discretion, depending on whether the patient benefits. During maintenance treatment, safety assessments and necessary tests will be performed every 1 month, investigators will be required to collect serious adverse events considered to be related to the study drug, oncologic assessments will be performed every 2 months to determine if the patient is appropriate to continue study treatment, and immunogenicity assessments will be performed every 2 months.

**Study population：**

**Patients need to meet all of the following criteria to receive study drug therapy**：

1) Age l8-70 years.

2) A diagnosis of colorectal cancer confirmed by pathological examination.

3) Metastatic colorectal cancer that has failed chemotherapy with fluorouracil, oxaliplatin and irinotecan.

4) Tumor tissue RAS gene wild type (including KRAS gene and NRAS gene).

5) The presence of measurable or non-measurable tumor lesions according to RECIST criteria version 1.1.

6) Expected survival of ≥ 3 months.

7) ECOG physical status score of 0 to 1.

8) No serious hematological, hepatic or renal abnormalities.

①Hematology: WBC > 4×109 /L, neutrophils > 1.5×l09 /L, PLT > 100×109 /L, HGB > 90 g/L;

②Liver function: glutamate transaminase and glutamic oxalacetic transaminase (ALT and AST < upper limit of normal value × 1.5 for those without liver metastases.

(ALT and AST < upper limit of normal value × 5), alkaline phosphatase (ALP < upper limit of normal value × 2.6 for those without liver metastases and ALP < upper limit of normal value × 5 for those with liver metastases), and total bilirubin (TBIL) < upper limit of normal value × 1.5.

③Renal function: creatinine (Cr) < upper limit of normal value × 1.5.

9) No history of other malignancies, except for cured cervical carcinoma in situ, basal or squamous cell carcinoma of the skin, epithelial tumors of the bladder, and surgically resected malignancies alone with disease free survival of ≥ 5 consecutive years.

10) No serious comorbidities (e.g., hypertension, coronary artery disease, diabetes, and history of mental illness).

11) Female subjects of childbearing age who have a negative pregnancy test and who voluntarily use effective and reliable contraception during the clinical trial.

12) Voluntarily participate in this clinical trial and sign the informed consent form.

**Patients are not eligible for study treatment if they meet any of the following criteria**：

1) Previous treatment with EGFR inhibitors, including EGFR TKI small molecule agents and anti-EGFR monoclonal antibodies.

2) Patients treated with antineoplastic therapy within the last 4 weeks (including corticosteroid therapy, within 6 weeks for patients who have received nitrosourea or mitomycin C) or patients who still have ≥2 degree toxicities due to prior antineoplastic therapy at the time of enrollment (CTCAE version 4.0).

3) Having undergone major surgery within 28 days, such as caesarean section, open-heart surgery, organ removal, etc.

4) Pre-existing interstitial lung disease (interstitial pneumonia or pulmonary fibrosis), or current imaging showing (on CT) interstitial lung disease.

5) An eye inflammation or eye infection that is not fully controlled, or any condition that could lead to one of these eye conditions.

6) Any unstable systemic disease: including but not limited to: active infection requiring systemic antibiotic therapy, congestive heart failure (New York Heart Association [NYHA] ≥ Class II, Appendix IX), angina pectoris, angioplasty, stenting, or myocardial infarction within 6 months prior to enrollment, hypertension not controlled by standard therapy, CTCAE 4.0 degree 3 or greater requiring pharmacologic therapy Arrhythmias, or asymptomatic sustained ventricular tachycardia, peripheral nerve disease of CTCAE version 4.0 degree 2 or greater.

7) HIV-positive, or active uncontrolled hepatitis.

8) Patients with documented and/or symptomatic brain metastases or soft meningeal metastases (a patient may be enrolled in this trial if at the time of enrollment the patient is assessed to be clinically stable for brain metastases, defined as being asymptomatic for 4 weeks prior to enrollment, while not requiring further treatment, such as radiation therapy, surgical resection, and/or hormonal therapy).

9) Allergies or known hypersensitivity to antibody-related components.

10) Have used or are using other experimental drug therapy within 4 weeks prior to treatment.

11) Prolonged use of drugs that interfere with the evaluation of test drug-related toxicity or immune activity (e.g., high doses [>20 mg/day] of prednisone analogs, high doses of NSAIDs).

12) Women who are pregnant or breastfeeding.

13) Patients who are known to have alcohol or drug addiction, or who, in the opinion of the investigator, have other adverse health conditions or mental status that may affect their assessment of protocol compliance and trial metrics and are not suitable for study participation.

**Dose and number of subjects administered.**

Single and sequential multiple dosing trials were conducted in six dose groups of 0.5 mg/kg, 1.0 mg/kg, 2.0 mg/kg, 4.0 mg/kg, 6.0 mg/kg and 8.0 mg/kg; subjects who completed the single dose safety and tolerability assessment were allowed to continue into multiple dosing trials at the original dose level after a 3-week washout period. Three to six patients were enrolled in each dose group for the single and multiple dosing trials. To obtain comparable data on pharmacokinetic parameters across dosing regimens, sequential multiple dosing trials allow for expanded enrollment at the target dose. Starting with version 5.0 of the protocol, the expanded enrollment regimen was revised to include SCT200 6.0 mg/kg administered weekly for 6 weeks, followed by 8.0 mg/kg administered every 2 weeks for continuous dosing. Subjects entering the maintenance phase (those who have completed SCT200 6.0 mg/kg for more than 6 weeks) should follow this revised regimen and subsequently receive 8.0 mg/kg every 2 weeks as a continuous dosing regimen. Therefore, the actual number of subjects participating in this trial will be determined by the dose-limiting toxicity (DLT) observed in the trial and the number of subjects in each dose group. Patients in the expanded enrollment group will not be involved in the DLT assessment.

New dose groups: 9.0 mg/kg/week, 12.0 mg/kg/week, 15.0 mg/kg/week, a total of 3 dose escalation groups, no single dose pharmacokinetic study was conducted in the new dose group. Under the additional doses of 9.0 mg/kg/week, 12.0 mg/kg/week and 15.0 mg/kg/week, patients were enrolled to receive the study drug sequentially from low dose to high dose according to the principle of dose escalation, and the pharmacokinetic study of multiple doses was also conducted. Three to six patients were enrolled in each dose group.

**Evaluation:**

Safety was assessed by adverse events, serious adverse events, physical examination, vital signs, clinical laboratory tests, immunogenicity evaluation and events leading to treatment discontinuation due to drug-related toxicity.

Blood samples were collected at study specific time points for pharmacokinetic analysis. Pharmacokinetic evaluation metrics include, but are not limited to, Cmax, Cmin, AUC_0-t_ , AUC_0-inf_ , t_1/2_ , clearance, and Vd.

The efficacy assessment observes the best objective response, response duration, disease stabilization time and disease progression time, and the evaluation refers to the content of ctDNA non-invasive individualized diagnosis and treatment genetic test to comprehensively analyze the patient's treatment.
