# Supplementary material for: Safety, pharmacokinetics and efficacy of SCT200, an anti-EGFR monoclonal antibody in patients with wild-type KRAS/NRAS/BRAF metastatic colorectal cancer: a phase I dose-escalation and dose-expansion study
Source: BMC Cancer. 2022 Oct 28;22:1104. doi: 10.1186/s12885-022-10147-9 (PMC9617324; doi:10.1186/s12885-022-10147-9)
Supplement: Supplementary file 2 — Additional file 2: Supplementary Table 1. Occurrence of adverse events associated with study drugs (overall incidence >10%). [file 12885_2022_10147_MOESM2_ESM.doc]

**Supplementary Table 1.** Occurrence of adverse events associated with study drugs (overall incidence >10%)

|  | 0.5 mg/kg (n=3) | | 1.0 mg/kg (n=3) | | 2.0 mg/kg (n=3) | | 4.0 mg/kg (n=4) | | 6.0 mg/kg QW (n=28) | | 6.0 mg/kg Q2W (n=3) | | 8.0 mg/kg (n=3) | | 9.0 mg/kg (n=3) | | 12.0 mg/kg (n=3) | | 15.0 mg/kg (n=3) | | Total n (statistics) (n=56) | |
| --- | --- | --- | --- | --- | --- | --- | --- | --- | --- | --- | --- | --- | --- | --- | --- | --- | --- | --- | --- | --- | --- | --- |
|  | Any grade | Grade ≥3 | Any grade | Grade ≥3 | Any grade | Grade ≥3 | Any grade | Grade ≥3 | Any grade | Grade ≥3 | Any grade | Grade ≥3 | Any grade | Grade ≥3 | Any grade | Grade ≥3 | Any grade | Grade ≥3 | Any grade | Grade ≥3 | Any grade | Grade ≥3 |
| Dermotoxicity | 0 | 0 | 1 (33.3) | 0 | 3 (100.0) | 1 (33.3) | 4 (100.0) | 2 (50.0) | 27 (96.4) | 5 (17.9) | 3 (100.0) | 0 | 3 (100.0) | 0 | 3 (100.0) | 0 | 3 (100.0) | 0 | 3 (100.0) | 1 (33.3) | 50 (89.3) | 9 (16.1) |
| Acneiform dermatitis | 0 | 0 | 1 (33.3) | 0 | 3 (100.0) | 1 (33.3) | 4 (100.0) | 2 (50.0) | 27 (96.4) | 2 (7.1) | 3 (100.0) | 0 | 3 (100.0) | 0 | 3 (100.0) | 0 | 3 (100.0) | 0 | 3 (100.0) | 0 | 50 (89.3) | 5 (8.9) |
| Paronychia | 0 | 0 | 0 | 0 | 0 | 0 | 0 | 0 | 10 (35.7) | 0 | 2 (66.7) | 0 | 0 | 0 | 1 (33.3) | 0 | 3 (100.0) | 0 | 3 (100.0) | 0 | 19 (33.9) | 0 |
| Dry skin | 0 | 0 | 0 | 0 | 0 | 0 | 1 (25.0) | 0 | 4 (14.3) | 1 (3.6) | 2 (66.7) | 0 | 2 (66.7) | 0 | 0 | 0 | 0 | 0 | 3 (100.0) | 1 (33.3) | 12 (21.4) | 2 (3.6) |
| Hypomagnesemia | 0 | 0 | 0 | 0 | 0 | 0 | 1 (25.0) | 0 | 24 (85.7) | 3 (10.7) | 3 (100.0) | 0 | 3 (100.0) | 0 | 3 (100.0) | 2 (66.7) | 3 (100.0) | 0 | 3 (100.0) | 0 | 40 (71.4) | 5 (8.9) |
| Elevated alanine aminotransferase | 0 | 0 | 2 (66.7) | 0 | 0 | 0 | 1 (25.0) | 0 | 9 (32.1) | 0 | 1 (33.3) | 0 | 1 (33.3) | 0 | 1 (33.3) | 0 | 1 (33.3) | 0 | 0 | 0 | 16 (28.6) | 0 |
| Hypertriglyceridemia | 0 | 0 | 1 (33.3) | 0 | 0 | 0 | 0 | 0 | 6 (21.4) | 0 | 3 (100.0) | 0 | 2 (66.7) | 0 | 1 (33.3) | 0 | 1 (33.3) | 0 | 0 | 0 | 14 (25.0) | 0 |
| Elevated aspartate aminotransferase | 0 | 0 | 2 (66.7) | 0 | 0 | 0 | 0 | 0 | 6 (21.4) | 0 | 0 | 0 | 1 (33.3) | 0 | 1 (33.3) | 0 | 0 | 0 | 0 | 0 | 10 (17.9) | 0 |
| Elevated blood bilirubin | 1 (33.3) | 0 | 2 (66.7) | 0 | 0 | 0 | 1 (25.0) | 0 | 2 (7.1) | 0 | 2 (66.7) | 0 | 1 (33.3) | 0 | 1 (33.3) | 0 | 0 | 0 | 0 | 0 | 10 (17.9) | 0 |
| Hypophosphatemia | 0 | 0 | 0 | 0 | 0 | 0 | 0 | 0 | 7 (25.0) | 0 | 1 (33.3) | 0 | 0 | 0 | 1 (33.3) | 0 | 1 (33.3) | 0 | 0 | 0 | 10 (17.9) | 0 |
| Conjunctivitis | 0 | 0 | 0 | 0 | 0 | 0 | 0 | 0 | 7 (25.0) | 0 | 1 (33.3) | 0 | 0 | 0 | 0 | 0 | 1 (33.3) | 0 | 0 | 0 | 9 (16.1) | 0 |
| Hypercholesterolemia | 0 | 0 | 3 (100.0) | 0 | 0 | 0 | 1 (25.0) | 0 | 3 (10.7) | 0 | 0 | 0 | 1 (33.3) | 0 | 0 | 0 | 0 | 0 | 0 | 0 | 8 (14.3) | 0 |
| Elevated blood alkaline phosphatase | 0 | 0 | 2 (66.7) | 0 | 1 (33.3) | 0 | 1 (25.0) | 0 | 1 (3.6) | 0 | 0 | 0 | 1 (33.3) | 0 | 0 | 0 | 0 | 0 | 0 | 0 | 6 (10.7) | 0 |
