# Supplementary material for: Safety, pharmacokinetics and efficacy of SCT200, an anti-EGFR monoclonal antibody in patients with wild-type KRAS/NRAS/BRAF metastatic colorectal cancer: a phase I dose-escalation and dose-expansion study
Source: BMC Cancer. 2022 Oct 28;22:1104. doi: 10.1186/s12885-022-10147-9 (PMC9617324; doi:10.1186/s12885-022-10147-9)
Supplement: Supplementary file 3 — Additional file 3: Supplementary Table 2. Kaplan–Meier method—time to first dermotoxicity/hypomagnesemia event. [file 12885_2022_10147_MOESM3_ESM.docx]

**Supplementary Table 2.** Kaplan–Meier method—time to first dermotoxicity/hypomagnesemia event

| Parameters  Statistical quantity | 0.5 mg/kg (n=3) | 1.0 mg/kg (n=3) | 2.0 mg/kg (n=3) | 4.0 mg/kg (n=4) | 6.0 mg/kg QW (n=28) | 6.0 mg/kg Q2W (n=3) | 8.0 mg/kg (n=3) | 9.0 mg/kg (n=3) | 12.0 mg/kg (n=3) | 15.0 mg/kg (n=3) | Overall (n=56) |
| --- | --- | --- | --- | --- | --- | --- | --- | --- | --- | --- | --- |
| Time to first dermotoxicity event (days) | | | | | | |  |  |  |  |  |
| Median (95% confidence interval) | N/A | N/A (50.0, N/A) | 8.0 (4.0, 11.0) | 7.5 (6.0, 13.0) | 7.0 (6.0, 9.0) | 6.0 (4.0, 7.0) | 8.0 (7.0, 9.0) | 5.0 (5.0, 10.0) | 5.0 (5.0, 11.0) | 10.0 (9.0, 14.0) | 8.0 (6.0, 9.0) |
| Time to first hypomagnesemia event (days) | | | | | | |  |  |  |  |  |
| Median (95% confidence interval) | N/A | N/A | N/A | N/A (63.0, N/A) | 43.0 (35.0, 78.0) | 67.0 (66.0, 103.0) | 60.0 (35.0, 66.0) | 35.0 (33.0, 35.0) | 42.0 (40.0, 77.0) | 28.0 (26.0, 47.0) | 66.0 (41.0, 78.0) |

Time to first event (days) was defined as the time between the start of the study treatment and the first event

N/A: not applicable
